# Prognostic Model to Predict Postoperative Adverse Events in Pediatric Patients With Aortic Coarctation

**DOI:** 10.3389/fcvm.2021.672627

**Published:** 2021-05-21

**Authors:** Yan Gu, Qianqian Li, Rui Lin, Wenxi Jiang, Xue Wang, Gengxu Zhou, Junwu Su, Xiangming Fan, Pei Gao, Mei Jin, Yuan Wang, Jie Du

**Affiliations:** ^1^Beijing Anzhen Hospital, Capital Medical University, Beijing, China; ^2^Key Laboratory of Remodeling-Related Cardiovascular Diseases, Ministry of Education, Beijing, China; ^3^Collaborative Innovation Centre for Cardiovascular Disorders, Beijing, China; ^4^Beijing Institute of Heart, Lung and Blood Vessel Diseases, Beijing, China; ^5^Department of Pediatric Cardiology, Beijing Anzhen Hospital, Capital Medical University, Beijing, China; ^6^Beijing Pediatric Heart Centre, Beijing, China; ^7^Department of Epidemiology and Biostatistics, School of Public Health, Peking University, Beijing, China; ^8^Department of Pediatric Cardiology, Bayi Children's Hospital Affiliated to the Seventh Medical Center of PLA General Hospital, Beijing, China; ^9^Peking University Clinical Research Institute, Peking University Health Science Center, Peking University, Beijing, China; ^10^Key Laboratory of Molecular Cardiovascular Sciences (Peking University), Ministry of Education, Beijing, China

**Keywords:** prediction, prognosis, risk stratification, coarctation of aorta, congenital heart disease

## Abstract

**Background:** Postoperative adverse events remain excessively high in surgical patients with coarctation of aorta (CoA). Currently, there is no generally accepted strategy to predict these patients' individual outcomes.

**Objective:** This study aimed to develop a risk model for the prediction of postoperative risk in pediatric patients with CoA.

**Methods:** In total, 514 patients with CoA at two centers were enrolled. Using daily clinical practice data, we developed a model to predict 30-day or in-hospital adverse events after the operation. The least absolute shrinkage and selection operator approach was applied to select predictor variables and logistic regression was used to develop the model. Model performance was estimated using the receiver-operating characteristic curve, the Hosmer–Lemeshow test and the calibration plot. Net reclassification improvement (NRI) and integrated discrimination improvement (IDI) compared with existing risk strategies were assessed.

**Results:** Postoperative adverse events occurred in 195 (37.9%) patients in the overall population. Nine predictive variables were identified, including incision of left thoracotomy, preoperative ventilation, concomitant ventricular septal defect, preoperative cardiac dysfunction, severe pulmonary hypertension, height, weight-for-age *z*-score, left ventricular ejection fraction and left ventricular posterior wall thickness. A multivariable logistic model [area under the curve = 0.8195 (95% CI: 0.7514–0.8876)] with adequate calibration was developed. Model performance was significantly improved compared with the existing Aristotle Basic Complexity (ABC) score (NRI = 47.3%, IDI = 11.5%) and the Risk Adjustment for Congenital Heart Surgery (RACHS-1) (NRI = 75.0%, IDI = 14.9%) in the validation set.

**Conclusion:** Using daily clinical variables, we generated and validated an easy-to-apply postoperative risk model for patients with CoA. This model exhibited a remarkable improvement over the ABC score and the RACHS-1 method.

## Introduction

Coarctation of aorta (CoA), which accounts for 6–8% of congenital heart disease (CHD), is a common disease with an incidence of about 1 in 2,500 live births (1–3). With advances in surgical technology, the peri-operative mortality has decreased to <3% (4, 5). However, the incidence of postoperative complications still remains high, at 36–68.8% (5, 6). There is no generally accepted tool to accurately predict the risk of postoperative adverse events individually in patients with CoA. Contemporary risk strategies for CHD mainly include the Aristotle Basic Complexity (ABC) score and the Risk Adjustment for Congenital Heart Surgery (RACHS-1), the Society of Thoracic Surgeons–European Association for Cardio-Thoracic Surgery Congenital Heart Surgery (STAT) mortality score and category, the STAT morbidity score and category. Such risk tools were developed on the basis of expert opinions or procedural complexity at the population-level, and focus primarily on in-hospital mortality or morbidity (6–10). Therefore, there is an unmet clinical need for comprehensive, individual assessment of CoA prognosis (10, 11).

In addition to surgical procedures, abnormal hemodynamics caused by structural malformation can lead to a series of complex pathophysiologic processes, such as hypertension, compensatory left ventricular hypertrophy, fibrosis, and cardiac insufficiency. These factors are known to contribute to the risks on mortality and morbidity (3, 12). Hence, the correlations between the patient specific influence factors and outcomes should be assessed.

Accordingly, we aimed to develop a risk model for the prediction of postoperative risk in pediatric patients with CoA, using daily clinical variables.

## Methods

**Figure 1:**
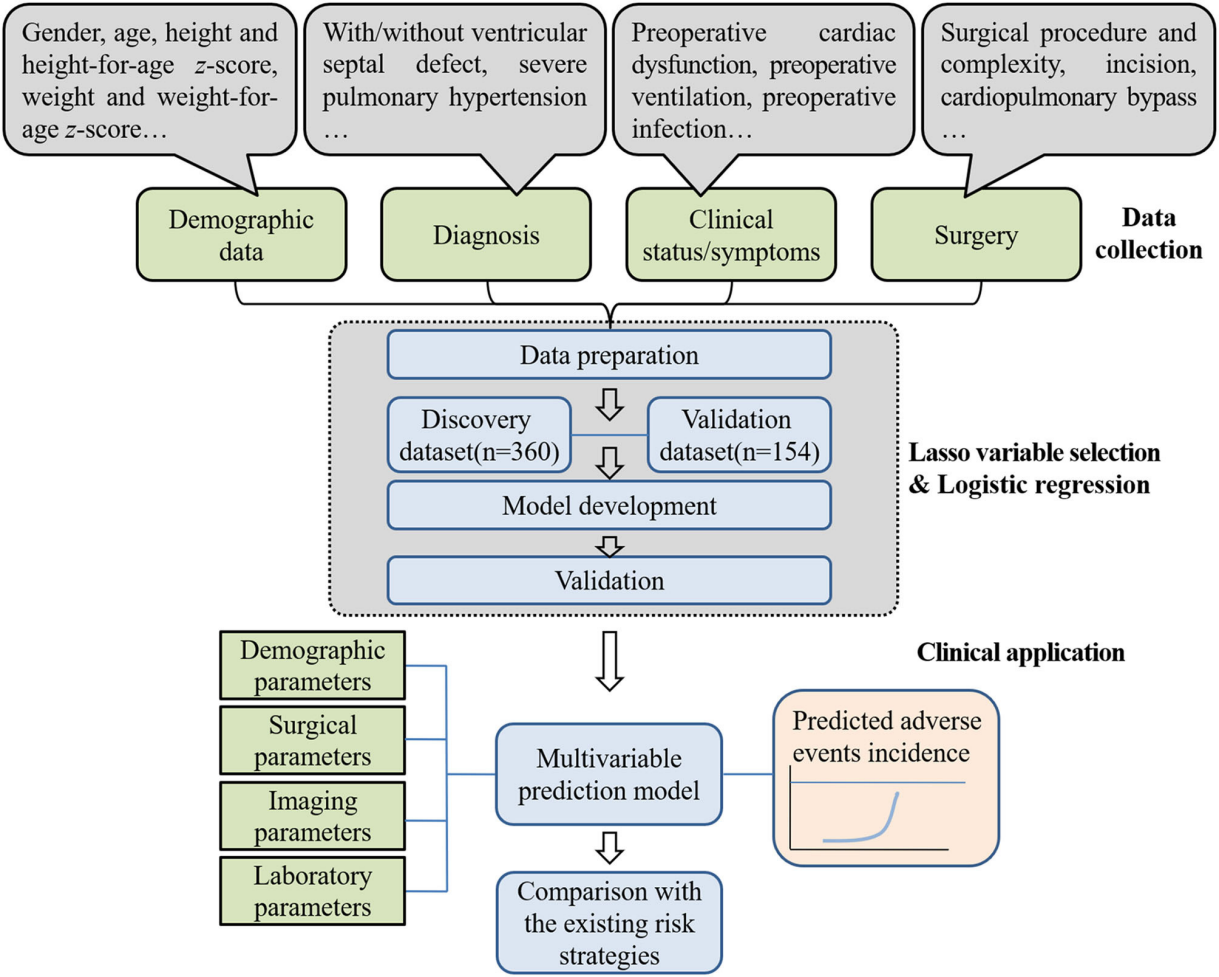
Overview of the phases we have followed to construct the predictive model. The Lasso method used clinical data input and selected predictor variables. In the end, a postoperative risk modeled for 30-day or in-hospital adverse events after operation was developed that including multiple variables contained prognostic information. The performance of the newly developed model was compared with that of the existing risk strategies. The speech bubbles illustrate the type of input used in the Lasso method. The input examples shown are from parts of the candidate variables.

Figure 1 shows the flow chart of the study process we have followed to build our predictive model, carry out the data collection, and conduct the model development and validation.

### Study Population

The data used to develop and validate the model came from the multi-center registration study of Prognosis and Integrative Assessment of Aortic Coarctation Patients in China (PICC) (ClinicalTrials.gov ID: NCT 04011956), and data on children undergoing CoA correction were retrospectively collected in the two centers: Center 1 (Beijing Anzhen Hospital, Beijing, China) from January 2002 to July 2020 and Center 2 (Bayi Children's Hospital affiliated to the Seventh Medical Center of PLA General Hospital, Beijing, China) from January 2012 to July 2020.

Patients were diagnosed by echocardiography and computed tomography, and they were scheduled for surgery according to the widely accepted indications for treatment (13–16). Given the informed consents of parents/legal guardians, we included patients who underwent CoA surgery in the two centers. Among all patients with CoA who underwent surgery at age≤17 years, we excluded those with complicated co-morbidities that could independently affect cardiovascular function, such as hypoplastic left heart syndrome, interruption of the aortic arch, Shone syndrome, and moderate or severe mitral stenosis, as well as patients with a history of known vasculopathy, genetic syndromes, diabetes mellitus, hyperlipoidemia or other cardiovascular risk factors. Patients with incomplete data on the variables of interest were also excluded.

### Outcomes

The aim of our study was to predict postoperative adverse events in patients with CoA undergoing surgery correction. Postoperative adverse events were defined as death and complication. Death was defined as death for any reason during hospitalization or after discharge within 30 days after operation (11, 17), and complications were defined as previously reported (18) and exampled in the Society of Thoracic Surgeons Congenital Heart Surgery Database. The main complications considered included low cardiac output, renal dysfunction or renal failure, cardiopulmonary resuscitation, ECMO assistance, pulmonary hypertensive crisis, postoperative infection, sepsis, chylothorax, pleural effusion requiring drainage, pericardial effusion, recurrent laryngeal nerve injury, diaphragm paralysis, unplanned thoracotomy during hospitalization or within 30 days after surgery, unplanned readmission, spinal cord injury, arrhythmia, and wound infection or poor healing (detailed categories and definition were listed in Supplementary Method 1).

### Data Collection

All clinical data were identified from electronic health records, including diagnosis, clinical status/symptoms, and operation performed. Data quality control was performed before the data analysis.

Data reflecting key pathophysiology obtained in routine clinical practice were included. Candidate variables included patient demographic and clinical characteristics, as well as imaging, surgical and laboratory variables. A total of 64 daily variables with <20% missing data that reflected the patient-specific characteristics or pathophysiological factors and were related to the prognosis were selected a priori based on the published data and clinical experiences (candidate variables and definitions are listed in Supplementary Method 2) (5, 6, 19–23).

### Statistical Analysis

Dataset from the Center 1 and the Center 2 were randomly separated into discovery set and validation set with a ratio of 7:3. There were 360 patients (70%) in the discovery set and 154 patients (30%) in the validation set. We used multiple imputation to handle the missing values. The number and proportion of missing data in discovery dataset were described in Supplementary Table 1. The majority of the variables had no missing information. We used 10 datasets for the multiple imputation. The model was trained in each imputed dataset. Common variables across the datasets were selected in the final model.

The least absolute shrinkage and selection operator (Lasso) regularization was used for variable selection. Stringent thresholds were used for retaining variables, which helped in the selection of a parsimonious, predictive subset of variables to train the logistic regression model, and the lambda parameter was calculated through three-fold cross-validation (the maximum value of λ is shown in Supplementary Figure 1). Then the screened variables with statistical significance (*p* < 0.05) in univariate logistic regression were further included as predictors to develop the multivariable model for assessing risk of outcomes with Stata software.

Receiver operating characteristic curves were used to estimate model discrimination by calculating the area under the curve (AUC). Calibration measured the agreement between the observed and predictive probabilities, and it was assessed by the Hosmer–Lemeshow test and calibration plot in this study. We defined five groups based on the quantiles of the predicted probabilities. In addition, net reclassification improvement (NRI) and integrated discrimination improvement (IDI) were used to demonstrate the improvement in the performance when comparing the developed model with the existing risk strategies. Moreover, we analyzed the predictor variables' effects using their odds ratio (OR) values and coefficients in the model. In addition, a nomogram was presented to predict the individual incidence of adverse events for each patient.

Performance was assessed in the validation set and overall study population, and sensitivity analyses were performed across subgroups by gender, age and RACHS-1 category in the datasets. Specifically, the datasets were divided into the following four age subgroups: ≤ 1month, 1–6 months, 6–12 months, and > 12 months. With regard to the RACHS-1 category, the patients were divided into three subgroups: category 1, category 2 or 3, and category 4. The prediction of adverse events was assessed to compare the models' performance in different subgroups.

Regarding the comparisons of the developed model and existing risk strategies, seven existing risk strategies, including the RACHS-1 method, the ABC score and category, the STAT mortality score and category, the STAT morbidity score and category, were applied as predictors of postoperative adverse events in the overall population (Supplementary Method 3).

For continuous variables, normally distributed variables were expressed as means ± standard deviations, and non-normally distributed variables were expressed as medians (interquartile ranges). Categorical variables were expressed as the frequencies or percentages. Continuous variables of two groups were compared by the bilateral independent *t*-test or the Wilcoxon test, and categorical variables were compared by chi-square test or Fisher's exact test and analyzed by Mann–Whitney *U*-test. Analyses were performed using Stata14.1 (StataCorp, College Station, Texas, USA), and *p* < 0.05 was considered statistically significant.

## Results

### Characteristics of the Study Population

There were a total of 514 patients with CoA correction enrolled in PICC study as of July 2020 in Center 1 (*n* = 360) and Center 2 (*n* = 154) (Supplementary Figure 2). The overall incidence of adverse events was 37.9% (*n* = 195), and there was a statistically significantly difference in this incidence between the two centers (30% for Center 1 and 56.5% for Center 2, *p* < 0.05). The Categories and incidence rates of adverse events in Center 1 and Center 2 were listed in Supplementary Table 2, showing that there were more than 30 categories of adverse events, with slightly different distributions in the two centers.

Demographic and clinical characteristics, imaging and surgical parameters, and laboratory values were compared between patients with and without adverse events (Table 1). Patients with adverse events were more likely to be younger, to be shorter in height, to weigh less, to have a lower left ventricular ejection fraction (LVEF), to have a history of preoperative ventilation, and to have a preoperative cardiac dysfunction. In addition, adverse events were associated with concomitant ventricular septal defect (VSD) and incision other than left thoracotomy, as well as a higher RACHS-1 category and a higher ABC score.

**Table 1:** Baseline clinical characteristics of CoA patients with and without adverse events (*N* = 514)^*^.

**Variables**	**Overall population**	**Adverse events**	**No adverse events**	***P***
	**(*N* = 514)**	**(*N* = 195)**	**(*N* = 319)**	
**Demographic variables**				
Median age, months (IQR)	5.0 (2.0, 13.0)	3.0 (1.0, 6.0)	7.0 (3.0, 24.0)	<0.0001
Median height, cm (IQR)	64.0 (55.0, 75.0)	59.0 (51.0, 66.0)	69.0 (60.0, 88.0)	<0.0001
Median weight, kg (IQR)	6.0 (4.2, 9.0)	4.8 (3.5, 6.3)	7.3 (5.0, 11.3)	<0.0001
Male, *n* (%)	317 (61.7)	113 (57.9)	204 (63.9)	0.1745
BMI, *z* score (SD)	−1.3 (1.8)	−1.7 (1.8)	−1.1 (1.7)	0.0006
Height-for-age, *z* score (SD)	−0.6 (1.9)	−0.9 (1.9)	−0.4 (1.8)	0.0025
Weight-for-age, *z* score (SD)	−1.3 (1.6)	−1.7 (1.7)	−1.1 (1.5)	<0.0001
**Clinical variables**				
History of heart failure, *n* (%)	19 (3.7)	5 (2.6)	14 (4.4)	0.2921
Median age at diagnosis, months (IQR)	1.0 (0.1, 5.0)	0.4 (0.1, 3.0)	2.0 (0.1, 8.0)	0.0001
Premature, *n* (%)	34 (6.8)	20 (10.6)	14 (4.4)	0.0074
Non-cardiac lesions, *n* (%)	23 (4.5)	11 (5.6)	12 (3.8)	0.3173
History of pneumonia, *n* (%)	189 (37.1)	62 (32.0)	127 (40.2)	0.0617
Median preoperative length of stay, days (IQR)	7.0 (4.0, 12.0)	7.0 (4.0, 13.0)	6.0 (3.0, 11.0)	0.1652
Preoperative ventilation, *n* (%)	58 (11.3)	38 (19.5)	20 (6.3)	<0.0001
Preoperative infection, *n* (%)	141 (27.4)	76 (39.0)	65 (20.4)	<0.0001
Preoperative systolic blood pressure, mmHg (SD)	103 (20)	96 (19)	107 (20)	<0.0001
Preoperative hypertension, *n* (%)	265 (51.6)	72 (36.9)	193 (60.5)	<0.0001
**Imaging variables**				
Concomitant VSD, *n* (%)	255 (49.7)	143 (73.7)	112 (35.1)	<0.0001
LVEF, % (SD)	66 (9)	64 (10)	68 (8)	<0.0001
Preoperative cardiac dysfunction, *n* (%)	45 (8.8)	26 (13.3)	19 (6.0)	0.008
Hypoplasia of aortic arch, *n* (%)	152 (29.7)	81 (42.0)	71 (22.3)	<0.0001
Bicuspid aortic valve, *n* (%)	49 (9.5)	13 (6.7)	36 (11.3)	0.0836
Preoperative pressure gradient, mmHg (SD)	47 (21)	41 (20)	51 (20)	<0.0001
Aortic isthmus diameter, mm (SD)	3.1 (1.2)	2.9 (1.0)	3.3 (1.2)	0.0006
Maximum velocity across isthmus, cm/s (SD)	330.8 (86.3)	304.6 (83.2)	345.6 (84.6)	<0.0001
Diameter of ascending aorta, mm (IQR)	10.0 (9.0, 13.4)	9.2 (8.0, 11.0)	11.0 (9.5, 15.0)	<0.0001
Diameter ratio of isthmus to ascending aorta, mean (SD)	0.3 (0.1)	0.3 (0.1)	0.3 (0.1)	0.0517
LVEDD, *z* score (SD)	1.1 (2.5)	1.2 (2.8)	1.1 (2.4)	0.8336
Severe PH, *n* (%)	208 (40.5)	119 (61.0)	89 (27.9)	<0.0001
Diameter ratio of pulmonary artery to ascending artery, (IQR)	1.3 (1.1, 1.7)	1.5 (1.2, 1.8)	1.3 (1.1, 1.6)	0.0026
EA ratio >1, *n* (%)	56 (10.9)	32 (16.4)	24 (7.5)	0.0017
Median IVS, cm (IQR)	0.6 (0.4, 0.7)	0.5 (0.4, 0.6)	0.6 (0.5, 0.7)	<0.0001
Median LVPW, cm (IQR)	0.5 (0.4, 0.6)	0.5 (0.4, 0.6)	0.6 (0.5, 0.7)	<0.0001
LVEDD, cm (SD)	2.8 (0.8)	2.6 (0.8)	2.9 (0.8)	<0.0001
Preoperative left ventricular mass, g (IQR)	31.5 (18.9, 48.0)	24.2 (12.7, 35.4)	36.2 (24.4, 53.2)	<0.0001
Preoperative left ventricular mass index, g/ m^2.7^ (IQR)	84.4 (59.5, 116.6)	88.8 (60.3, 118.7)	83.0 (58.8, 115.0)	0.2308
Preoperative left ventricular hypertrophy, n (%)	330 (65.5)	111 (59.0)	219 (69.3)	0.0191
Concomitant myocardial abnormality, *n* (%)	31 (6.0)	9 (4.6)	22 (6.9)	0.2918
Relative wall thickness, (SD)	0.4 (0.1)	0.4 (0.1)	0.4 (0.1)	0.1924
Left ventricular remodeling, *n* (%)	397 (78.8)	144 (76.6)	253 (80.1)	0.3573
**Surgical variables**				
Incision of left thoracotomy, *n* (%)	258 (50.2)	52 (26.7)	206 (64.6)	<0.0001
Cardiopulmonary bypass, *n* (%)	249 (48.4)	142 (72.8)	107 (33.5)	<0.0001
^†^Surgical procedure type, *n* (%)				<0.0001
1	113 (22.0)	24 (12.3)	89 (27.9)	
2	100 (19.5)	23 (11.8)	77 (24.1)	
3	97 (18.9)	51 (26.2)	46 (14.4)	
4	116 (22.6)	73 (37.4)	43 (13.5)	
5	88 (17.1)	24 (12.3)	64 (20.1)	
RACHS-1, *n* (%)				<0.0001
1	230 (44.7)	42 (21.5)	188 (58.9)	
2	36 (7.0)	16 (8.2)	20 (6.3)	
3	115 (22.4)	64 (32.8)	51 (16.0)	
4	133 (25.9)	73 (37.4)	60 (18.8)	
ABC score, (IQR)	8.0 (6.0, 10.0)	10.0 (7.0, 10.0)	6.0 (6.0, 10.0)	<0.0001
**Laboratory variables**				
AST, U/L (IQR)	40.0 (32.0, 52.0)	42.0 (32.0, 55.0)	39.0 (32.0, 49.0)	0.0264
median NLR, (IQR)	0.6 (0.3, 1.1)	0.6 (0.4, 1.3)	0.5 (0.3, 1.0)	0.0331
Leucocytes, × 10^9^/L (SD)	9.6 (3.6)	10.0 (4.3)	9.3 (3.1)	0.0342
Lymphocyte, × 10^9^/L (SD)	5.0 (2.1)	4.9 (2.2)	5.1 (2.1)	0.2673
Neutrophil, × 10^9^/L (IQR)	2.8 (2.0, 4.1)	2.8 (1.9, 4.8)	2.8 (2.0, 3.8)	0.0035
ALT, U/L (IQR)	20.0 (14.0, 29.0)	22.0 (15.0, 30.0)	20.0 (14.0, 29.0)	0.1027
CK, U/L (IQR)	112.0 (78.0, 176.0)	110.0 (77.0, 184.0)	113.0 (79.0, 174.0)	0.1013
Monocyte, × 10^9^/L (IQR)	0.6 (0.4, 0.8)	0.6 (0.4, 0.9)	0.5 (0.4, 0.7)	0.0017
Hemoglobin, g/L (SD)	117.6 (20.8)	116.8 (21.4)	118.0 (20.5)	0.5331
PLT, × 10^9^/L (SD)	306.8 (104.4)	307.7 (98.9)	305.2 (113.1)	0.7937
Red blood cell, × 10^9^/L (SD)	4.2 (0.7)	4.1 (0.7)	4.3 (0.7)	0.0003
Urea, mmol/L (IQR)	3.7 (2.6, 4.8)	3.7 (2.5, 5.2)	3.6 (2.6, 4.7)	0.1466
Red cell volume distribution width, % (SD)	14.5 (2.8)	14.8 (2.2)	14.3 (3.1)	0.0244
Creatine, μmol/L (SD)	31.7 (22.0)	34.4 (18.1)	30.1 (23.9)	0.0355
Uric acid, μmol/L (SD)	262.0 (104.7)	276.4 (125.8)	253.5 (89.0)	0.0182
Glucose, mmol/L (SD)	4.8 (1.5)	4.8 (1.6)	4.8 (1.4)	0.7733
Triglyceride, mmol/L (IQR)	0.9 (0.6, 1.3)	0.9 (0.7, 1.3)	0.9 (0.6, 1.2)	0.1469
Total cholesterol, mmol/L (SD)	3.5 (0.9)	3.3 (1.0)	3.7 (0.9)	<0.0001
HDL cholesterol, mmol/L (SD)	1.2 (0.9)	1.2 (1.3)	1.2 (0.4)	0.3216

* *For continuous variables, non-normally distributed variables are expressed as the median (IQRs), normally distributed variables are expressed as means (SDs). Categorical variables are presented as n (%). P < 0.05 was considered statistically significant*.

† *Surgical procedure was coded as 1 for end-to-end anastomosis for patients with isolated aortic coarctation (CoA) except patent ductus arteriosus (PDA), 2 for non-end-to-end anastomosis for patients with isolated CoA, 3 for CoA correction with ventricular septal defect (VSD) repair in patients with VSD; 4 for hypoplasia of aortic arch (HAA) correction with VSD repair in patients with VSD, and 5 for CoA correction with pulmonary artery banding or PDA ligation. VSD, ventricular septal defect; LVEF, left ventricular ejection fraction; LVEDD, left ventricular end-diastolic dimension; PH, pulmonary hypertension; IVS, interventricular septal thickness; LVPW, left ventricular posterior wall thickness; ABC, Aristotle Basic Complexity; RACHS-1, Risk Adjustment for Congenital Heart Surgery; NLR, neutrophil-to-lymphocyte ratio*.

### Predictor Variables and Model Derivation

There was no significant difference between the discovery set and the validation set in most of the demographic factors and clinical characteristics such as age, gender, weight, height, preoperative ventilation, preoperative cardiac dysfunction, surgical procedure type, incision, left ventricular remodeling, or in the RACHS-1 or ABC score (*p* > 0.05). This finding indicated that most of the baseline clinical features of the patients were similar between the discovery set and validation set (Supplementary Table 1).

Using Lasso followed by univariate logistic regression, nine variables of surgical factors, patient-specific and pathophysiological factors were identified, such as incision of left thoracotomy, preoperative ventilation, concomitant VSD, preoperative cardiac dysfunction, severe pulmonary hypertension (PH), height, weight-for-age *z*-score, LVEF and left ventricular posterior wall thickness (LVPW). And the final Lasso combined with logistic regression model (the Lasso model) was developed by these nine predictor variables.

**Figure 2:**
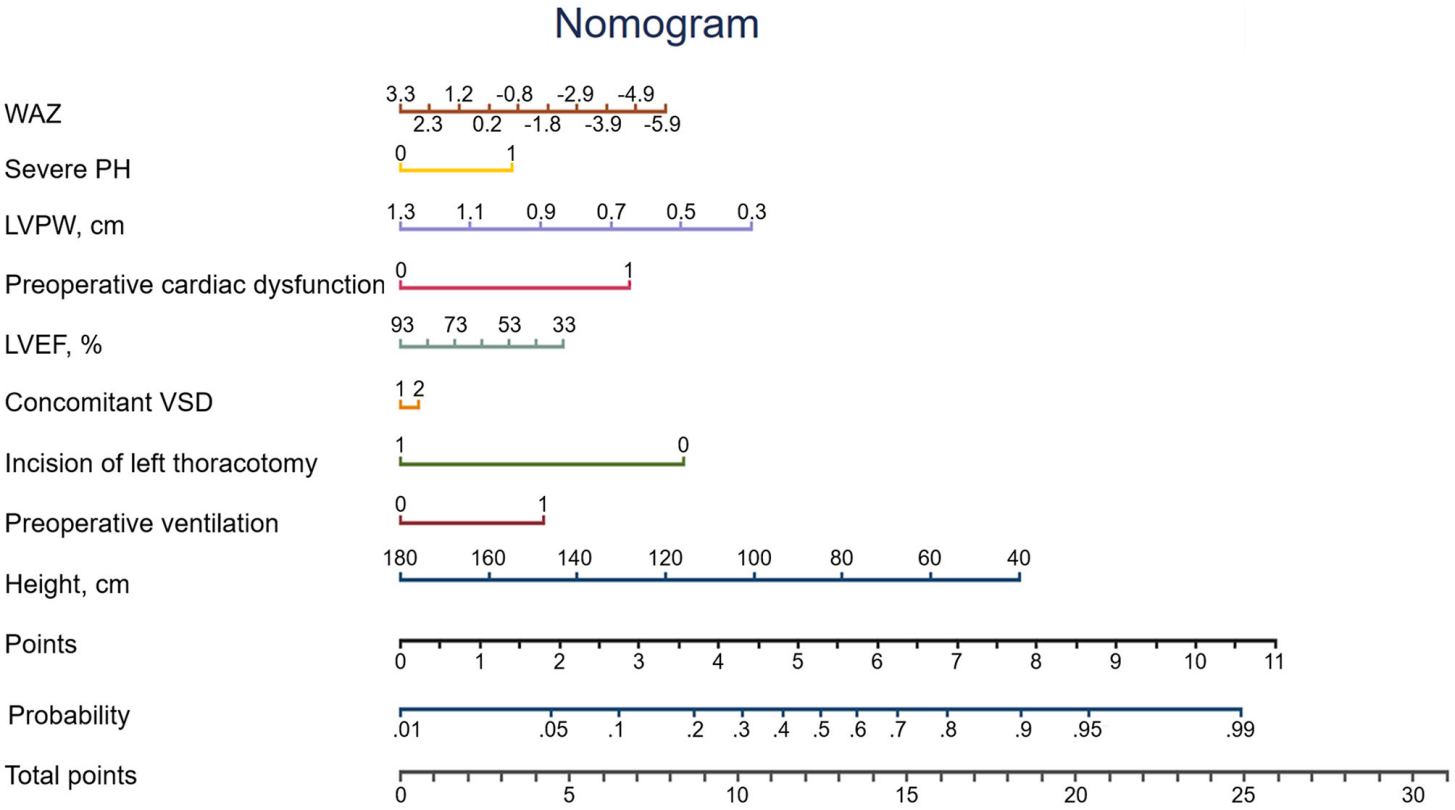
Nomogram of the newly developed Lasso model.

The missing values for the predictor variables in the discovery set, the validation set and the overall population were demonstrated in Supplemental Table 3, the beta coefficients and ORs of the variables in the Lasso model were shown in Table 2. The adjusted OR values of the four variables—preoperative cardiac dysfunction, preoperative ventilation, severe PH, and concomitant VSD—were > 1 (2.908, 1.939, 1.684, and 1.087, respectively). The top five variables in terms of the high absolute value of their coefficients were LVPW, left thoracotomy incision, preoperative cardiac dysfunction, preoperative ventilation and severe PH. A nomogram was constructed according to the regression coefficients in the model (Figure 2) for easily and better clinical use, such as in information sharing and decision-making for both clinicians and patients.

**Table 2:** Beta coefficients and odds ratios of the newly developed Lasso model.

**Variables**	**Crude OR**	95% CI^*^	**Adjusted OR**	**95% CI^**†**^**	**β-coefficient**
Height	0.959	0.944–0.973	0.980	0.958–1.002	−0.020
Preoperative ventilation	4.283	2.147–8.543	1.939	0.837–4.491	0.662
Incision of left thoracotomy	0.208	0.131–0.328	0.269	0.128–0.565	−1.313
Concomitant VSD	5.104	3.214–8.106	1.087	0.493–2.396	0.083
LVEF	0.954	0.932–0.978	0.988	0.949–1.028	−0.012
Preoperative cardiac dysfunction	2.143	1.029–4.461	2.908	0.880–9.610	1.067
LVPW	0.014	0.003–0.069	0.212	0.027–1.661	−1.551
Severe PH	3.358	2.148–5.247	1.684	0.920–3.082	0.521
WAZ	0.758	0.659–0.871	0.875	0.736–1.040	−0.134
Intercept term			42.869	1.230–1494.287	

*Lasso, least absolute shrinkage and selection operator; OR, odds ratio; CI, confidence interval; WAZ, Weight-for-age z-score; ABC, Aristotle Basic Complexity; PH, pulmonary hypertension; VSD, ventricular septal defect; LVEF, left ventricular ejection fraction; LVPW, left ventricular posterior wall thickness*.

* *95% confidence interval of crude OR;*

† *95% confidence interval of adjusted OR*.

### Model Performance

**Figure 3:**
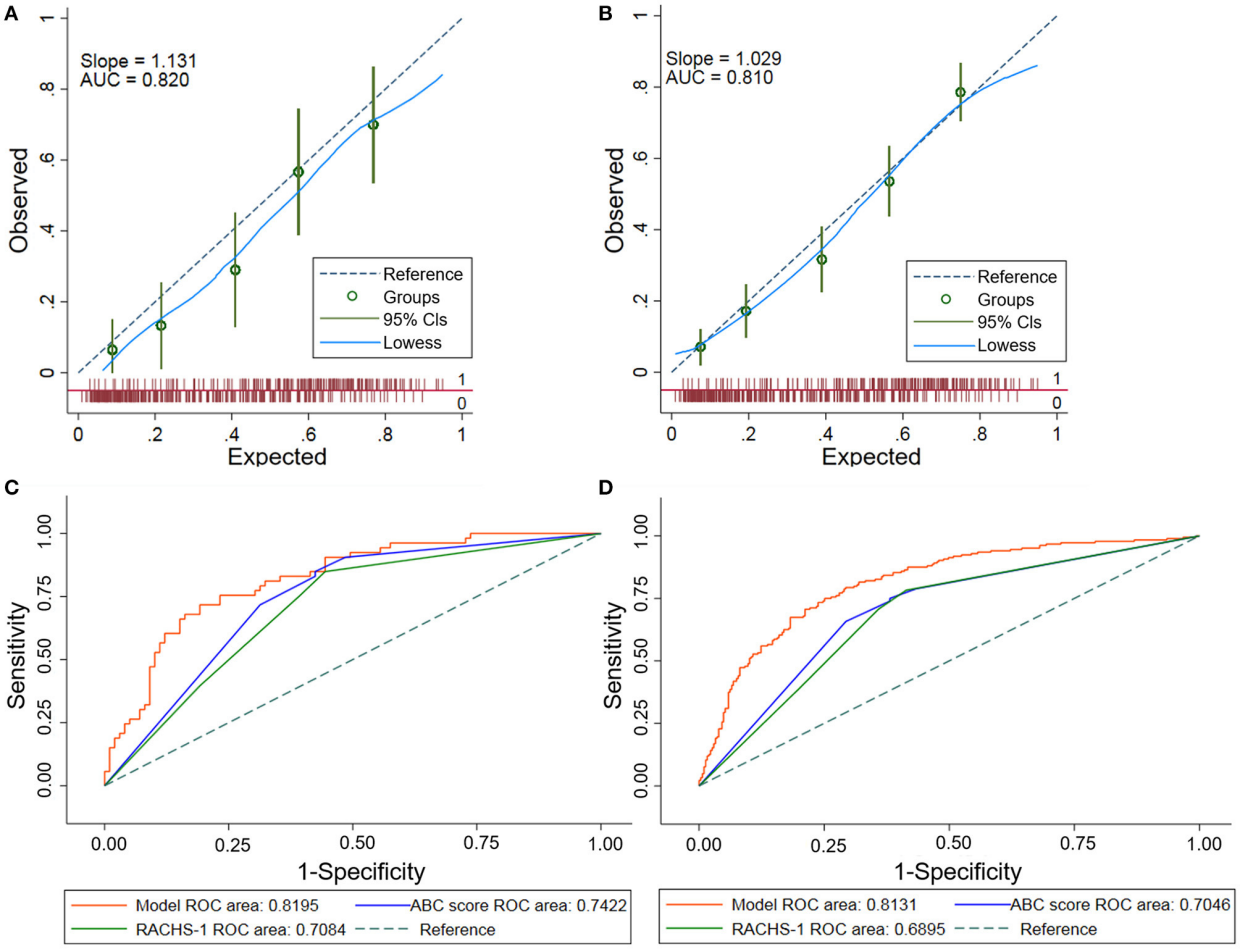
Performance assessments of the Lasso model. **(A)** Calibration plot of the Lasso model in the validation set. The dashed diagonal line represents perfect calibration. Observed adverse events are shown with the corresponding 95% CIs. The *x-*axis is the predicted probability estimated by the model and the *y-*axis is the actual probability. **(B)** Calibration plot of the Lasso model in the overall population. There was a good agreement between the predicted and observed probabilities in the overall population. The dashed diagonal line represents perfect calibration. Observed adverse events are shown with the corresponding 95% CIs. **(C)** Receiver operating characteristic (ROC) curves comparing the Lasso model with the ABC score and the RACHS-1 in validation set. This comparison showed that the Lasso model outperformed the ABC score and the RACHS-1 in prediction of postoperative adverse events in the validation set (*p* < 0.05). **(D)** Receiver operating characteristic (ROC) curves comparing the Lasso model with the ABC score and the RACHS-1 in the overall population. The Lasso model outperformed the ABC score and the RACHS-1 in prediction of postoperative adverse events in the overall population (*p* < 0.001). Lasso, least absolute shrinkage and selection operator; ABC, Aristotle Basic Complexity; RACHS-1, Risk Adjustment for Congenital Heart Surgery.

The final Lasso model achieved an AUC of 0.8195 (95%CI: 0.7514–0.8876). Calibration of the model, assessed using the Hosmer–Lemeshow chi-square test in the validation set, indicated adequate calibration (goodness-of-fit *p* = 0.257). The corresponding predicted and observed adverse event rates according to the model were detailed in Figure 3A, which showed slightly higher predicted than observed rates.

The model calibrated well in the overall population using the Hosmer–Lemeshow test (goodness-of-fit *p* = 0.307**)**. Good calibration was also shown by the closeness of the line fitting the data to the diagonal line in the calibration plot (Figure 3B). This finding indicated an agreement between the predicted probabilities and the actual observations.

In addition, to test the model's performance in a broader set of patients, the postoperative adverse events were assessed in subgroups divided by gender, age, and RACHS-1 category in the discovery set, validation set and the overall population, respectively (shown in Supplementary Figure 3). The AUCs in the subgroups were consistent with each other in all datasets (*p* > 0.05), which suggested the ability of the model to predict outcomes across different gender, age and RACHS-1 category subgroups. Moreover, given the potential discrepancy between predicted probabilities and outcomes in the validation set, a stratified analysis with measures of calibration as observed/expected ratios was also conducted in the overall study population. The results showed that the observed/expected ratios in the gender- and RACHS-1 category-specific subgroups were close to one, and that the ratios deviated from one in the age-specific subgroups, especially for children older than 6 months (Supplementary Table 4).

### Comparisons With the Current Risk Strategies

There were no statistical difference in the AUCs among the seven current clinically used risk strategies (0.6851–0.7050, *p* = 0.11) (Supplementary Table 5). Hence, two commonly used methods (the ABC score and RACHS-1) were adopted for further comparisons with the newly developed Lasso model. The AUC for the Lasso model (0.8195) was greater than that for either the ABC score (0.7422) or the RACHS-1 method (0.7084) in the validation set (Figure 3C) (*p* < 0.05 for each comparison with the Lasso model). In addition, model performances in terms of reclassification and discrimination was significantly improved compared with the ABC score (NRI = 47.3%, IDI = 11.5%) and the RACHS-1(NRI = 75.0%, IDI = 14.9%) (Table 3).

**Table 3:** Improved model performance over the existing ABC score and RACHS-1 method.

	**Validation set**		**Overall population**	
**Model**	**ABC score^**a**^**	**RACHS-1^**b**^**	**ABC score^**a**^**	**RACHS-1^**b**^**
NRI	0.473	0.750	0.708	0.744
95% CI^*^	0.142–0.804	0.419–1.081	0.530–0.886	0.566–0.922
IDI	0.115	0.149	0.130	0.153
95% CI^†^	0.058–0.172	0.088–0.210	0.100–0.161	0.121–0.185

*ABC, Aristotle Basic Complexity; RACHS-1, Risk Adjustment for Congenital Heart Surgery; NRI, net reclassification improvement; IDI, integrated discrimination improvement*.

a *performance improvement compared with ABC score;*

b *performance improvement compared with RACHS-1 method*.

* *95% confidence interval of NRI;*

† *95% confidence interval of IDI*.

The Lasso model was superior to both of the ABC score and the RACHS-1 category when these models were applied to the overall population (AUC = 0.8131, 0.7046, and 0.6895, respectively; *p* < 0.001) (Figure 3D). Moreover, remarkable improvements in reclassification and discrimination was achieved in the overall population, comparing the Lasso model with the ABC score (NRI = 70.8%, IDI = 13.0%) and with the RACHS-1(NRI = 74.4%, IDI=15.3%) (Table 3).

## Discussion

To the best of our knowledge, this is the first study based on a large multi-center pediatric CoA cohort in China. Predictor variables from routine clinical data were identified to generate a risk prediction model.

The obstructive structural malformation of CoA can lead to a series of abnormal pathophysiology changes, such as hemodynamic abnormalities, compensatory left ventricular hypertrophy, left ventricular remodeling, subendocardial myocardial hypoperfusion, ischemia, fibrosis, and cardiac dysfunction. With the correction of the anatomical malformation and hemodynamics, left ventricular remodeling occurs, and ventricular morphology and function are improved (24, 25). Variations and variances in these processes lead to different postoperative outcomes for individual patients.

In this study, we combined the death and complications as the outcome for several reasons. First, although death and complications are adverse events of differing severity, there are many commonalities in mechanisms and pathologies, which are mainly related to three categories of factors: individual factors, operation-related factors and pathological factors. In this study, the multivariable model also reflects the impact of different factors on the outcome events. Second, as the postoperative adverse events in our composite outcomes are heterogeneous, with scattered specific outcomes and different detailed classifications due to the large sample size, it is not feasible to accurately evaluate the predictive effectiveness of the model for each outcome in an individual-level analysis.

To better reflect prognoses at the individual level compared with the currently used methods, patient-specific and pathophysiologic variables were screened using the Lasso method. Given the combined advantages of ridge regression and subset selection (26), the Lasso method has better filtering capability compared with stepwise selection (27) and can better identify the key factors influencing postoperative prognosis to generate interpretable models. In addition, logistic regression models are commonly used in the field of medical research. Therefore, we chose these methods for selecting the variables and constructing the risk model.

The developed nine-variable Lasso model was well-validated and showed **s**ignificant improvement over existing risk strategies for postoperative prediction in patients with CoA. Moreover, the identified predictor variables reflecting patient-specific and pathophysiological factors were interpretable. First, the incision variable was added to reflect the surgical effects on the postoperative prognosis. Repair of CoA through left thoracotomy has been shown to have low risks of perioperative morbidity and mortality (28). The protective role is partly because of its associated isolated CoA and non-CPB conditions, which have been shown to be associated with a reduction in some adverse consequences (29). Second, preoperative ventilation has been identified as a patient-specific risk factor for patients of different ages (19) that, when combined with severe PH, may reflect the status of pulmonary circulation, perfusion, vascular resistance and function, when combined with the variable of severe PH. Therefore, the risk of various pulmonary complications (such as pulmonary infection, pneumothorax, atelectasis, respiratory insufficiency resulting in repeated intubations, and tracheotomy) will increase with preoperative ventilation. Furthermore, severe PH is thought to be associated with restrictive left ventricular physiology, accompanied by left ventricular endocardial fibrosis, and is an independent risk factor for death and readmission of heart failure (30). Third, the presence of VSD is associated with increased left ventricular volume load and pulmonary perfusion resulting from abnormal hemodynamics (31). Furthermore, median sternotomy is indicated in patients who have one-stage correction of concomitant VSD, and median sternotomy increases the risk of postoperative complications such as bleeding, chylothorax, and atelectasis (32). Fourth, as both the left ventricular systolic and diastolic function have been found to be impaired to different degrees among patients with CoA (33), and preoperative LVEF reflects the reserved function of the left ventricle, cardiac dysfunction indicated the state of decompensation. Even if structural malformations and hemodynamic abnormalities are corrected by the operation, reverse remodeling of the left ventricle would be incomplete, resulting in postoperative adverse events such as low cardiac output and pericardial effusion (34). Fifth, variables of patient-specific factors such as height, weight-for-age *z*-score reflect preoperative growth, nutritional status, and perfusion of systemic circulation, so a deficiency in height and weight indicates a high risk of death and other postoperative adverse events (35, 36). Finally, the variable of LVPW reflects the status of pre-operation left ventricular remodeling, but this variable was discovered also to be related to other factors such as diagnosis group (LVPW was higher in the group without VSD), age (to a certain extent reflecting the course of disease for congenital malformation), and severe PH (LVPW was greater in the non-severe PH group than in the severe PH group) in this study. In view of the above, predictor variables included in the Lasso model reflect the combined effects of the systemic pathological states and should be interpreted in an integrated manner.

Regarding the variables' weights and contributions to the model, it could be inferred from the coefficients that the variables with relatively high contribution to the model were LVPW, incision of left thoracotomy, preoperative cardiac dysfunction, preoperative ventilation and severe PH, which reflected the inherent pathophysiology including ventricular remodeling, surgery, cardiac load and function, and had good concordance with clinical findings.

In addition, we noticed that an overestimation occurred among patients with a middle level of risk in the validation dataset. We further compared the baseline characteristics of patients in middle range of the risk group between discovery and validation datasets to explore the possible reasons for this overestimation. We found that the proportion of female, as well as the levels of ALT, hemoglobin and relative wall thickness were higher in the validation dataset than in the discovery dataset (Supplementary Table 6), suggesting the existence of a difference in the populations of the discovery and validation datasets. Further validation is still needed to assess the model performance, especially among Western populations.

### Strengths

The main strength of our study was that, compared with the existing risk strategies, the newly developed model was able to provide better prediction of outcomes in patients with CoA by incorporating pathophysiological factors. For better understanding and more objective comparison, two postoperative evaluation methods that are commonly used in clinical practice (the ABC score and the RACHS-1 method) were applied as comparisons. Although the ABC score and the RACHS-1 method are mainly designed to evaluate the risk of death, they also reflect the state of being at high risk for postoperative complications. Our newly developed model is aimed at the composite outcome of death and complications, potentially contributing to the reduction in risks of both complications and death.

The most important issue for a clinical risk model is how it can help the clinicians and benefit the patients. Regarding our newly developed model, first, using the clinical variables collected preoperatively, an individual patient's risk of adverse events can be assessed by the model or the associated nomograph. Patients can also be informed regarding their risk factors and the incidence rates of adverse events in detail before the operation to facilitate thorough communication and appropriate decision-making together with the clinicians, which will contribute to avoiding unpredictable medical disputes later in the process. Second, our newly developed model could also be served as a tool in the identification of high-risk patients, allowing clinicians to optimize monitoring and the selection of treatment strategies, and reducing adverse events.

Nevertheless, further efforts toward precision medicine are warranted, such as either defining outcomes as certain subgroup of complications, or detecting circulatory biomarkers as an additional screening tool in risk stratification. This was an exploratory study and we hope to expand the sample size and to perform prospective research to make the findings more generalizable and applicable for clinical use in the future.

### Limitations

This study had several limitations. First, the data used in this study derived from two centers located in the same city; therefore the established model may not be generalizable to other centers. However, the patient population was representative, and the total sample size was relatively large. Second, the data were collected retrospectively across a relatively long time-span to attain a large sample size, it is possible that the weight of some of the risk factors considered into the model may have changed over time, and limited daily variables were available in the database. A better prediction model might be established by adding more variables and applying a different modeling approach. Third, an overestimation occurred among patients with a middle level of risk in the validation sample, which indicated the existence of a difference in the populations in the discovery and validation datasets, and further validation is still needed to assess model performance. Fourth, given that there was no existing evaluation method for the postoperative composite outcome of CoA, we chose relatively classical methods that are widely used in clinical application but that were not initially developed for the purpose of comparison; because of this, the comparison is not completely “fair”.

## Conclusion

Using daily clinical variables, we generated and validated a postoperative risk model for pediatric patients with CoA. With significantly improved performance over the existing risk strategies to which it was compared, the newly developed model could be served as a tool for preoperative risk stratification and contribute to the reduction of adverse events.

## Data Availability Statement

The original contributions presented in the study are included in the article/Supplementary Materials, further inquiries can be directed to the corresponding author/s.

## Ethics Statement

This study was approved by the Ethics Review Board of Beijing Anzhen Hospital and Bayi Children's Hospital affiliated to the Seventh Medical Center of PLA General Hospital, and all informed consents of parents/legal guardians were obtained.

## Author Contributions

YG conceptualized the study, carried out the analyses, and drafted the initial manuscript. YW and MJ conceptualized the study, supervised the analyses, and reviewed and revised the manuscript. JD conceptualized the study, supervised the analyses, reviewed and revised the manuscript, and was responsible for the administration of PICC registry study. QL conducted data collection and statistical analysis. PG supervised the analyses, and reviewed the manuscript. RL, WJ, and XW collected the data and conducted the literature search. GZ, JS, and XF conceptualized the study and reviewed the manuscript. All authors were involved in the manuscript review, and agree to be accountable for the content of the work.

## Conflict of Interest

The authors declare that the research was conducted in the absence of any commercial or financial relationships that could be construed as a potential conflict of interest.
