# Supplementary material for: Prognostic Model to Predict Postoperative Adverse Events in Pediatric Patients With Aortic Coarctation
Source: Front Cardiovasc Med. 2021 May 21;8:672627. doi: 10.3389/fcvm.2021.672627 (PMC8175771; doi:10.3389/fcvm.2021.672627)
Supplement: Supplementary file 1 [file Data_Sheet_1.pdf]

Supplementary Material

Supplementary Methods

Supplementary Method 1. Adverse events and definitions (1)

(1). Death due to any reason during hospitalization or after discharge within 30 days after operation. (2). Readmission is defined as any unplanned readmission to the hospital within 30 days of surgery or intervention. (3). Cardiac dysfunction is defined as LVEF < 50% post-operation. (4). Low cardiac output is characterized by some of the following: tachycardia, oliguria, decreased skin perfusion, need for increased inotropic support (10% above baseline at admission), metabolic acidosis, widened Arterial–Venous oxygen saturation. (5). ECMO assistance is defined as utilization of postoperative/postprocedural mechanical support-ECMO. (6). Pericardial effusion is defined as abnormal accumulation of fluid in the pericardial space, requiring medical management or drainage. (7). Pulmonary hypertensive crisis is an acute state of inadequate systemic perfusion associated with pulmonary hypertension. (8). Cardiopulmonary resuscitation is defined as postoperative cardiopulmonary resuscitation for any reason, ie. cardiac arrest. (9). Arrhythmia is defined as any cardiac rhythm other than Normal Sinus Rhythm (non-NSR), requiring medical or surgical treatment. (10). Pneumonia is defined as a respiratory disease characterized by inflammation of the lung parenchyma (including alveolar spaces and interstitial tissue). Pneumonia is diagnosed by appropriate clinical findings (such as fever, leukopenia or leukocytosis, and new onset of purulent sputum) and one or more of the following: positive cultures (of sputum or pulmonary secretions) and/or pulmonary infiltrate on chest X-ray. (11). Pneumothorax is a collection of gas in the pleural space resulting in collapse of the lung on the affected side. (12). Atelectasis is defined as the collapse of part or all of a lung by blockage of the bronchus or bronchioles leading to retraction of the lung, which is diagnosed by chest radiographs and/or CT scans. (13). Chylothorax is presence of lymphatic fluid in the pleural space, commonly secondary to leakage from the thoracic duct or one of its main tributaries. (14). Pleural effusion is defined as abnormal accumulation of fluid in the pleural space, requiring drainage. (15). Reintubation is defined as it is required after initial extubation. In other words, the need to reinstitute postoperative or postprocedural mechanical ventilation after a planned extubation and prior to discharge, or after a planned extubation and after discharge but within 30 days of surgery. (16). Pulmonary hemorrhage is defined as hemorrhage in alveoli or interstitium or both. The diagnosis needs comprehensive clinical, pulmonary imaging and laboratory examination. (17). Renal dysfunction or renal failure is defined as acute renal dysfunction or failure, requiring dialysis or hemofiltration. (18). Infection or Sepsis is defined as infection at any site (except pneumonia and wound infection) and sepsis. (19). Paralyzed diaphragm is presence of elevated hemi-diaphragm(s) on chest radiograph in conjunction with evidence of weak, immobile, or paradoxical movement assessed by ultrasound or fluoroscopy. (20). Seizure is defined as new on-set one occurring postoperatively and/or post-procedurally (prior to hospital discharge or after hospital discharge but less than 30 days after surgery and/or intervention). (21). Injury to recurrent laryngeal nerve is defined as intraoperative/Intraprocedural injury to recurrent laryngeal nerve. (22). Subarachnoid hemorrhage is the existence of a neurologic imaging study indicating a new or previously unsuspected collection of subarachnoid hemorrhage. (23). Subdural hemorrhage is defined as the existence of a neurologic imaging study indicating a new or previously unsuspected collection of subdural hemorrhage. (24). Peptic ulcer is defined as new on-set postoperatively, requiring medical

management. (25). Ascite is defined as ascite requiring paracentesis or placement of peritoneal drain. (26). Dysfunction of the liver that results in hypoalbuminemia (< 2 grams/dL), coagulopathy (PT > 1.5×upper limits of normal), and hyperbilirubinemia (> 3.0×upper limits of normal). It is defined if the patient develops 2 out of these 3 laboratory abnormalities. (27). Wound infection is defined as erythema, possible induration and possible fluctuance of a surgical wound (surgical site) with possible drainage and possible tissue separation, wound dehiscence is defined as separation of the layers of a surgical wound. (28). Sternum left open is defined as leaving the sternum open postoperatively (planned or unplanned) with the goal for delayed sternotomy closure. (29). Cardiac reoperation is defined as reoperation performed postoperative for any reason, ie. bleeding, tamponade. (30). Spinal cord injury newly acquired or newly recognized deficit of spinal cord function indicated by physical exam findings, imaging studies, or both. (31). Residual stenosis and restenosis are defined as residual or recurrent aortic arch gradient, requiring reoperation or intervention.

Supplementary Method 2. Candidate variables and definitions

(1). Demographic variables: gender, age, height and height-for-age z-score, weight and weight-for-age z-score, BMI z-score;(2) .Clinical variables: age of diagnosis, history of heart failure, premature, non-cardiac lesion, history of pneumonia, preoperative length of stay, preoperative ventilation; preoperative infection, preoperative systolic blood pressure and hypertension;(3) .Imaging variables: concomitant ventricular septal defect (VSD), preoperative left ventricular ejection fraction (LVEF), preoperative cardiac dysfunction, hypoplasia of aortic arch (HAA), bicuspid aortic valve (BAV), preoperative pressure gradient, aortic isthmus diameter, maximum velocity across isthmus, diameter of ascending aorta, diameter ratio of isthmus to ascending aorta, EA ratio greater than 1, interventicular septal thickness (IVS), left ventricular end diastolic diameter (LVEDD) and z-score of LVEDD, severe pulmonary hypertension (PH), diameter ratio of pulmonary artery to ascending artery, left ventricular posterior wall thickness (LVPW) preoperative left ventricular mass (LVM), preoperative left ventricular mass index (LVMI), preoperative left ventricular hypertrophy (LVH), concomitant myocardial abnormality relative wall thickness (RWT), left ventricular remodeling (LVR).(4).Surgical variables: incision of left thoracotomy, surgical procedure type, cardiopulmonary bypass, the Risk Adjustment for Congenital Heart Surgery (RACHS-1) category and the Aristotle Basic Complexity (ABC) score;(5).Laboratory variables: white blood cell (WBC), neutrophil count, lymphocyte count, neutrophil-to-lymphocyte (NLR), Monocyte count, alanine amino transferase (ALT), aspartate aminotransferase (AST), creatine kinase (CK), red blood cell (RBC), red cell volume distribution width (RDW), hemoglobin (Hb), platelet (PLT), triglyceride (TG), cholesterol (CHO), high-density lipoprotein (HDL), blood urea nitrogen (BUN), serum creatinine (SCr), uric acid (UA), and glucose (Glu).

Variables are defined as follows: Preoperative cardiac dysfunction was defined as a LVEF <50% before surgery (2, 3). Severe PH is defined as pulmonary artery systolic pressure (PASP)>60mmHg, and for children younger than 3 months by combining clinical symptoms and indirect ultrasound signs, such as pure right to left shunt at atrial level or ductus arteriosus level, paradoxical interventricular septal motion at end-systole. (4,5). HAA is defined as the literature (6). LVM and LVMI was calculated and LVH was defined as LVMI >95th percentile for gender due to the o the age-specific reference as mentioned (7, 8), and hypertension (HPT) was defined as greater than the 95th percentile based on sex, age and height on at least three separate measurements (9, 10). RWT was calculated by using the formula (IVS +LVPW)/LVEDD, we defined as LVR if the left ventricular geometry was classified as concentric (elevated LVMI and RWT), concentric remodeling (normal LVMI and elevated RWT), eccentric (elevated LVMI and normal RWT), according to the

literatures (11, 12). *Z* scores of weight and height were calculated using the World Health Organization (WHO) Child Growth Standards and Growth Reference data with the WHO anthropometric calculator, AnthroPlus v.1.0.4. *Z* score of LVEDD was calculated using the on-line tool of the Children's Hospital Boston (https://zscore.chboston.org/). Concomitant myocardial abnormality in this study was defined as positive when echocardiography showed (1). thickening of the endocardium by layers of collagenous and elastic fibres to >20 μm with enhanced echo (13), or (2). prominent trabeculations and deep intertrabecular recesses in the myocardium of the left ventricle (14).

Supplementary Method 3. Current risk strategies for congenital heart disease (CHD)

The RACHS-1 method was created with the clinical expertise of a nationally representative 11-member panel of experts, by which different congenital heart disease operations were divided into six risk levels and verified in the pediatric cardiac care Consortium database. Because a small amount of individual information is involved, it is suitable for the prediction of patient groups, but not for predicting individual death risk—it can only be used to assess in-hospital mortality but not other outcomes, such as complications and long-term mortality (15).

The ABC score was proposed by the Surgical Expert Group of Congenital Heart Disease from the Society of Thoracic Surgeons (STS) and the European Association for cardiothoracic surgery (EACTS) in 1999. According to potential mortality, complications, and technical difficulty of 145 kinds of congenital heart disease surgery, the operation was graded and divided into four risk levels. However, this score only considers simple pathology related to the surgical procedure, and is based on the main procedure rather than diagnosis, which is only applicable to the evaluation of the surgical procedure (16).

The STS–EACTS Congenital Heart Surgery (STAT) including Mortality Scores and Categories are an empirically based tool that statistically estimates the risk of mortality associated with operations for congenital heart disease, which was proposed by O'Brien in 2009. It was firstly named STS–EACTS Congenital Heart Surgery Mortality Score (or, briefly, the STS–EACTS score), and the STS–EACTS Congenital Heart Surgery Mortality Categories (or, briefly, the STS–EACTS categories) respectively. According to the mortality rates of 148 surgical procedures in 77,294 patients with congenital heart disease in the STS and EACTS databases, the operation was divided into five risk levels according to surgical mortality. However, it does not include the patient's personal complications and other related information, which is mainly used for the prediction of surgical mortality and the grading of the same level of surgery (17, 18). And STAT Morbidity Scores and Categories were developed in 2013, and firstly named STS Congenital Heart Surgery Morbidity Scores and Categories. In this scoring system, a total of 140 procedures were assigned scores ranging from 0.1 to 5.0 and sorted into 5 relatively homogeneous categories. The range was chosen to be the same as the existing STAT Mortality Score (19).

Supplementary Tables

Supplementary Table1.Baseline features of the discovery set and validation set*

Variables	Total (N=514)	Discovery set (N=360)	Missing values[#]	Validation set (N=154)	*p*
**Demographic variables**					
Median age, months (IQR)	5.0(2.0,13.0)	5(2.0-12.5)	0	5.0(2.0,13.0)	0.5395
Median height, cm (IQR)	64.0(55.0,75.0)	64.0(55.0,75.5)	0	64.0(55.0,74.0)	0.6234
Median weight, kg (IQR)	6.0(4.2,9.0)	6.1(4.3-9.4)	1(0.3)	6.0(4.0,8.5)	0.4899
Male, n (%)	317 (61.7)	220(61.1)	0	97(63.0)	0.6886
BMI, *z* score (SD)	-1.3(1.8)	-1.3(1.8)	1(0.3)	-1.4(1.7)	0.4658
Height-for-age, *z* score (SD)	-0.6(1.9)	-0.6(1.9)	0	-0.7(1.8)	0.6595
Weight-for-age, *z* score (SD)	-1.3(1.6)	-1.3(1.6)	9(2.5)	-1.4(1.6)	0.3992
**Clinical variables**					
Adverse events	195(37.9)	141(39.2)	0	54(35.1)	0.3800
Median age at diagnosis, months (IQR)	1.0(0.1,5.0)	1.0(0.1,5.0)	0	1.1(0.1,6.0)	0.2839
History of heart failure, n (%)	19(3.7)	15(4.2)	0	4(2.6)	0.3944
Premature, n (%)	34(6.8)	24(6.9)	10(2.8)	10(6.5)	0.895
Non-cardiac lesions, n (%)	23(4.5)	16(4.4)	0	7(4.5)	0.9595
History of pneumonia, n (%)	189(37.1)	127(35.6)	3(0.8)	62(40.5)	0.289
Median preoperative length of stay, days (IQR)	7.0(4.0,12.0)	7.0(4.0,12.0)	0	7.0(4.0,12.0)	0.8346
Preoperative ventilation, n (%)	58(11.3)	43(11.9)	0	15(9.7)	0.4694
Preoperative infection, n (%)	141(27.4)	106(29.4)	0	35(22.7)	0.1179
Preoperative systolic blood pressure, mmHg (SD)	103(20)	103(21)	0	103(19.6)	0.9369
Preoperative hypertension, n (%)	265(51.6)	184(51.1)	0	81(52.6)	0.7574
**Imaging variables**					
Concomitant VSD, n (%)	255(49.7)	172(47.9)	1(0.28)	83(53.9)	0.214
LVEF, % (SD)	66(9)	67(9)	0	66(9)	0.4124
Preoperative cardiac dysfunction, n (%)	45(8.8)	32(8.9)	0	13(8.4)	0.8694
Hypoplasia of aortic arch, n (%)	152(29.7)	107(29.8)	1(0.3)	45(29.4)	0.929
Bicuspid aortic valve, n (%)	49(9.5)	32(8.9)	0	17(11.0)	0.4470
Preoperative pressure gradient, mmHg (SD)	47(21)	49(21)	29(8)	45(20)	0.0762
Aortic isthmus diameter, mm (SD)	3.1(1.2)	3.2(1.2)	0	3.0(1.1)	0.2156
Maximum velocity across stenosis, cm/s (SD)	330.8(86.3)	335.7(84.5)	27(7.5)	319.2(89.5)	0.0568
Diameter of ascending aorta, mm (IQR)	10.0(9.0,13.4)	10.0(9.0,14.0)	5(1.4)	10.0(8.8,12.5)	0.3928
Diameter ratio of isthmus to ascending aorta, mean (SD)	0.3(0.1)	0.3(0.1)	5(1.4)	0.3(0.1)	0.4942
LVEDD, *z* score (SD)	1.1(2.5)	1.0(2.5)	10(2.8)	1.4(2.6)	0.1258
Severe PH, n (%)	208(40.5)	137(38.1)	0	71(46.1)	0.0886

Diameter ratio of pulmonary artery to ascending artery, (IQR)	1.3(1.1,1.7)	1.3(1.1,1.6)	4(1.1)	1.4(1.1,1.8)	0.0965
EA ratio greater than 1, n (%)	56(10.9)	36(10.0)	0	20(13.0)	0.3194
Median IVS, cm (IQR)	0.6(0.4,0.7)	0.6(0.4,0.7)	9(2.5)	0.6(0.4,0.7)	0.5993
Median LVPW, cm (IQR)	0.5(0.4,0.6)	0.5(0.4,0.6)	9(2.5)	0.5(0.4,0.6)	0.7605
LVEDD, cm (SD)	2.8(0.8)	2.8(0.8)	9(2.5)	2.8(0.8)	0.6794
Preoperative left ventricular mass, g (IQR)	31.5(18.9,48.0)	31.5(19.0-46.7)	9(2.5)	31.6(17.7,48.5)	0.9739
Preoperative LVMI, g/ $m^{2.7}$(IQR)	84.4(59.5, 116.6)	84.5(59.2-116.0)	9(2.5)	83.3(59.9,119.2)	0.2290
Preoperative left ventricular hypertrophy, n (%)	330(65.5)	232(66.1)	9(2.5)	98(64.1)	0.6571
Concomitant myocardial abnormality, n (%)	31(6.0)	17(4.7)	0	12(7.8)	0.167
Relative wall thickness, (SD)	0.4(0.1)	0.4(0.1)	9(2.5)	0.4(0.1)	0.4157
Left ventricular remodeling, n (%)	397(78.8)	280(79.8)	9(2.5)	117(76.5)	0.4046
**Surgical variables**					
Incision of left thoracotomy, n (%)	258(50.2)	189(52.5)	0	69(44.8)	0.1100
Cardiopulmonary bypass, n (%)	249(48.4)	168(46.7)	0	81(52.6)	0.2178
†Surgical procedure type, n (%)			0		0.3173
1	113(22.0)	86(23.9)		27(17.5)	
2	100(19.5)	66(18.3)		34(22.1)	
3	97(18.9)	62(17.2)		35(22.7)	
4	116(22.6)	83(23.1)		33(21.4)	
5	88(17.1)	63(17.5)		25(16.2)	
RACHS-1, n (%)			0		0.6028
1	230(44.7)	166(46.1)		64(41.6)	
2	36(7.0)	26(7.2)		10(6.5)	
3	115(22.4)	75(20.8)		40(26.0)	
4	133(25.9)	93(25.8)		40(26.0)	
ABC score, (IQR)	8.0(6.0,10.0)	7.0(6.0,10.0)	2(0.6)	8.0(6.0,10.0)	0.1443
**Laboratory variables**					
AST, U/L (IQR)	40.0(32.0,52.0)	39.0(31.0,49.0)	12(3.3)	43.0(33.5,55.0)	0.099
median NLR, (IQR)	0.6(0.3,1.1)	0.6(0.3,1.0)	9(2.5)	0.6(0.4,1.1)	0.5136
Leucocytes, x10^9/L (SD)	9.6(3.6)	9.6(3.7)	3(0.8)	9.6(3.5)	0.9822
Lymphocyte, x10^9/L (SD)	5.0 (2.1)	5.0(2.1)	3(0.8)	5.1(2.1)	0.5675
Neutrophil, x10^9/L(IQR)	2.8(2.0,4.1)	2.8(1.9-4.2)	3(0.8)	2.8(2.1,4.0)	0.8960
ALT, U/L (IQR)	20.0(14.0,29.0)	20.0(14.0,28.0)	9(2.5)	21.0(15.0,35.0)	0.0069
CK, U/L (IQR)	112.0(78.0, 176.0)	111.5(77.0, 174.0)	14(3.9)	112.0(79.0, 186.0)	0.7467
Monocyte, x10^9/L (IQR)	0.6(0.4,0.8)	0.6(0.4,0.8)	6(1.7)	0.6(0.4,0.8)	0.7746
Hemoglobin, g/L (SD)	117.6(20.8)	116.9(19.6)	2(0.6)	119.1(23.5)	0.2847
PLT, x10^9/L (SD)	306.8(104.4)	309.6(104.9)	2(0.6)	300.2(103.2)	0.3517
Red blood cell, x10^9/L (SD)	4.2(0.7)	4.2(0.7)	3(0.8)	4.3(0.7)	0.1084
Urea, mmol/L (IQR)	3.7(2.6,4.8)	3.7(2.6,4.8)	9(2.5)	3.6(2.5,5.0)	0.6489
Red cell volume distribution width, (SD)	14.5(2.8)	14.6(3.1)	5(1.4)	14.3(1.8)	0.2156
Creatine, µmol/L (SD)	31.7(22.0)	32.5(24.0)	10(2.8)	30.0(16.1)	0.2575

Uric acid, μmol/L (SD)	262.0(104.7)	263.6(105.4)	11(3.1)	258.3(103.3)	0.6060
Glucose, mmol/L(SD)	4.8(1.5)	4.8(1.6)	11(3.1)	4.8(1.1)	0.9097
Triglyceride, mmol/L (IQR)	0.9(0.6,1.3)	0.9(0.6,1.3)	23(6.4)	0.9(0.6,1.2)	0.4052
Total cholesterol, mmol/L (SD)	3.5(0.9)	3.5(1.0)	22(6.1)	3.5(0.9)	0.9243
HDL cholesterol, mmol/L (SD)	1.2(0.9)	1.2(1.0)	24(6.7)	1.2(0.3)	0.3900

* For continuous variables, non-normally distributed variables are expressed as the median (IQRs), normally distributed variables are expressed as means (SDs). Categorical variables are presented as *n* (%). $p < 0.05$ was considered statistically significant. # Missing values in the discovery set, variable with more than 3% missing values were multiple imputed. †Surgical procedure was coded as 1 for end-to-end anastomosis for patients with isolated aortic coarctation (CoA) except patent ductus arteriosus (PDA), 2 for non-end-to-end anastomosis for patients with isolated CoA, 3 for CoA correction with ventricular septal defect (VSD) repair in patients with VSD; 4 for hypoplasia of aortic arch (HAA) correction with VSD repair in patients with VSD and 5 for CoA correction with pulmonary artery banding or PDA ligation. VSD, ventricular septal defect; LVEF, left ventricular ejection fraction; LVEDD, left ventricular end-diastolic dimension; PH, pulmonary hypertension; IVS, interventicular septal thickness; LVPW, left ventricular posterior wall thickness; ABC, Aristotle Basic Complexity; RACHS-1, Risk Adjustment for Congenital Heart Surgery; NLR, neutrophil-to-lymphocyte ratio.

Supplementary Table 2. Categories and incidences of adverse events in the Center 1 and the Center 2

Adverse events	Center1	Center 2
Death	8(2.2%)	10(6.5%)
Readmission	4(1.1%)	2(1.3%)
Cardiac dysfunction	14(3.9%)	3(1.9%)
Low cardiac output	20(0.6%)	7(4.5%)
ECMO assistance	0	0
Pericardial effusion	5(1.4%)	1(0.6%)
Pulmonary hypertensive crisis	0	1(0.6%)
Low cardiac output +ECMO	0	1(0.6%)
Cardiac dysfunction + Pericardial effusion	1(0.3%)	0
Cardiopulmonary resuscitation	1(0.3%)	1(0.6%)
Arrhythmia	10(2.8%)	2(1.3%)
Pneumonia	22(6.1%)	6(3.9%)
Pneumothorax	4(1.1%)	3(1.9%)
Atelectasis	5(1.4%)	4(2.6%)
Chylothorax	6(1.7%)	4(2.6%)
Pleural effusion	9(2.5%)	0
Reintubation	0	3(1.9%)
Pneumonia + Atelectasis	5(1.4%)	1(0.6%)
Pneumonia + Reintubation	1(0.3%)	0
Pneumonia + Tracheotomy	1(0.3%)	0
Chylothorax + Pneumothorax	0	1(0.6%)
Pneumothorax + Reintubation	0	1(0.6%)

Pulmonary hemorrhage	0	1(0.6%)
Renal dysfunction/ Renal failure	0	6(3.9%)
Sepsis/Infection	9(2.5%)	6(3.9%)
Paralyzed diaphragm	1(0.3%)	0
Seizure	0	2(1.3%)
Injury to recurrent laryngeal nerve	0	1(0.6%)
Subarachnoid hemorrhage	1(0.3%)	0
Subdural hemorrhage	0	1(0.6%)
Peptic ulcer	0	1(0.6%)
Ascite	6(1.7%)	0
Liver dysfunction	0	2(1.3%)
Wound infection/ Wound dehiscence	4(1.1%)	2(1.3%)
Sternum left open	7(1.9%)	45(29.2%)
Cardiac reoperation	4(1.1%)	1(0.6%)
Others	1(0.3%)	2(1.3%)

Supplementary Table 3. Missing predictor variables of the model in discovery set, validation set and overall population

Variables	Overall population (N=514)	Discovery set (N=360)	Validation set (N=154)
Height, n (%)	0	0	0
Preoperative ventilation, n (%)	0	0	0
Incision of left thoracotomy, n (%)	0	0	0
Concomitant VSD, n (%)	1(0.19)	1(0.28)	0
LVEF, n (%)	0	0	0
Preoperative cardiac dysfunction, n (%)	0	0	0
LVPW, n (%)	10(1.95)	9(2.50)	1(0.65)
Severe PH, n (%)	0	0	0
WAZ, n (%)	10(1.95)	9(2.50)	1(0.65)

VSD, ventricular septal defect; LVEF, left ventricular ejection fraction; LVPW, left ventricular posterior wall thickness; PH, pulmonary hypertension; WAZ, Weight-for-age *z*-score.

Supplementary Table 4. Predicted and observed risk of postoperative adverse events in pediatric patients with aortic coarctation by 5th of predicted risk in the overall study population

Variables	Number of patients	Predicted risk	Observed risk	Ratio of predicted to observed (95%CI)
**Gender**				
Female	197	0.43	0.42	1.03 (0.68,1.38)
Male	317	0.37	0.36	1.04 (0.67,1.42)
**Mon**				

Mon ≤ 1	101	0.58	0.62	0.93 (0.66,1.20)
1 < mon ≤ 6	198	0.47	0.45	1.05 (0.71,1.38)
6 < mon ≤ 12	86	0.33	0.27	1.24 (0.74,1.73)
Mon > 12	129	0.17	0.16	1.12 (0.47,1.77)
**RACHS-1**				
1	230	0.20	0.18	1.08 (0.33,1.83)
2-3	151	0.55	0.53	1.04 (0.78,1.31)
4	133	0.56	0.55	1.01 (0.76,1.27)

RACHS-1, Risk Adjustment for Congenital Heart Surgery.

Supplementary Table 5. Prediction performances of the existing risk strategies for postoperative adverse events in overall population

Risk strategies	AUC	95% CI*	OR	95% CI†
RACHS-1	0.6938	0.6497-0.7379	1.810	1.554-2.107
ABC Score	0.7050	0.6623-0.7476	1.557	1.401-1.730
ABC Category	0.6974	0.6548-0.7400	2.325	1.894-2.852
STAT Mortality Score	0.6933	0.6476-0.7390	3.272	2.354-4.549
STAT Mortality Category	0.6851	0.6396-0.7306	1.817	1.540-2.144
STAT Morbidity Score	0.6905	0.6432-0.7378	2.503	1.962-3.194
STAT Morbidity Category	0.6935	0.6478-0.7393	2.030	1.676-2.458

AUC, area under the curve; CI, confidence interval; OR, odds ratio; RACHS-1, Risk Adjustment for Congenital Heart Surgery; ABC, Aristotle Basic Complexity; STAT, the Society of Thoracic Surgeons-European Association for Cardio-Thoracic Surgery Congenital Heart Surgery. *, 95% confidence interval of AUC; †, 95% confidence interval of OR.

Supplementary Table 6. Comparison of baseline characteristics between discovery and validation dataset of patients in middle range of the risk group

Variables	Discovery dataset (N=67)	Validation dataset (N=31)	*p*
Female, n (%)	21(31.3%)	16(51.6%)	0.0542
ALT, U/L (IQR)	19.0(14.0,25.0)	23.0(16.0,49.0)	0.0155
Hb, g/L (SD)	112.5(17.1)	120.5(14.8)	0.0287
Severe PH, n (%)	20(29.9%)	13(41.9%)	0.2391
Median height, cm (IQR)	63.0(56.0,70.0)	63.0(52.0,69.0)	0.5072
Weight-for-age, *z* score (SD)	-1.7(1.4)	-1.5(1.6)	0.4497
Preoperative ventilation, n (%)	5(7.5%)	5(16.1%)	0.1875
Incision of left thoracotomy, n (%)	36(53.7%)	17(54.8%)	0.9185

Concomitant VSD, n (%)	35(52.2%)	18(58.1%)	0.5904
LVEF, % (SD)	64.2(11.0)	63.7(8.1)	0.8162
Preoperative cardiac dysfunction, n (%)	14(20.9%)	3(9.7%)	0.1726
Median LVPW, cm (IQR)	0.5(0.5,0.6)	0.5(0.4,0.6)	0.9523

PH, pulmonary hypertension; WAZ, Weight-for-age *z*-score; VSD, ventricular septal defect; LVEF, left ventricular ejection fraction; LVPW, left ventricular posterior wall thickness.

Supplementary Figures

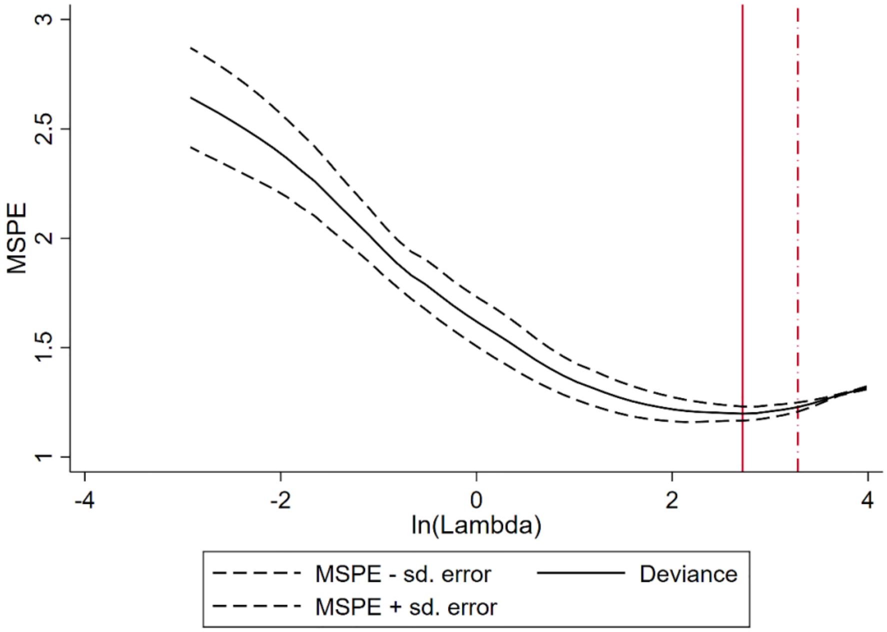

Supplementary Figure 1. The maximum value of λ within one standard deviation of the minimum mean square prediction error (MPSE) when selecting variables by Lasso.

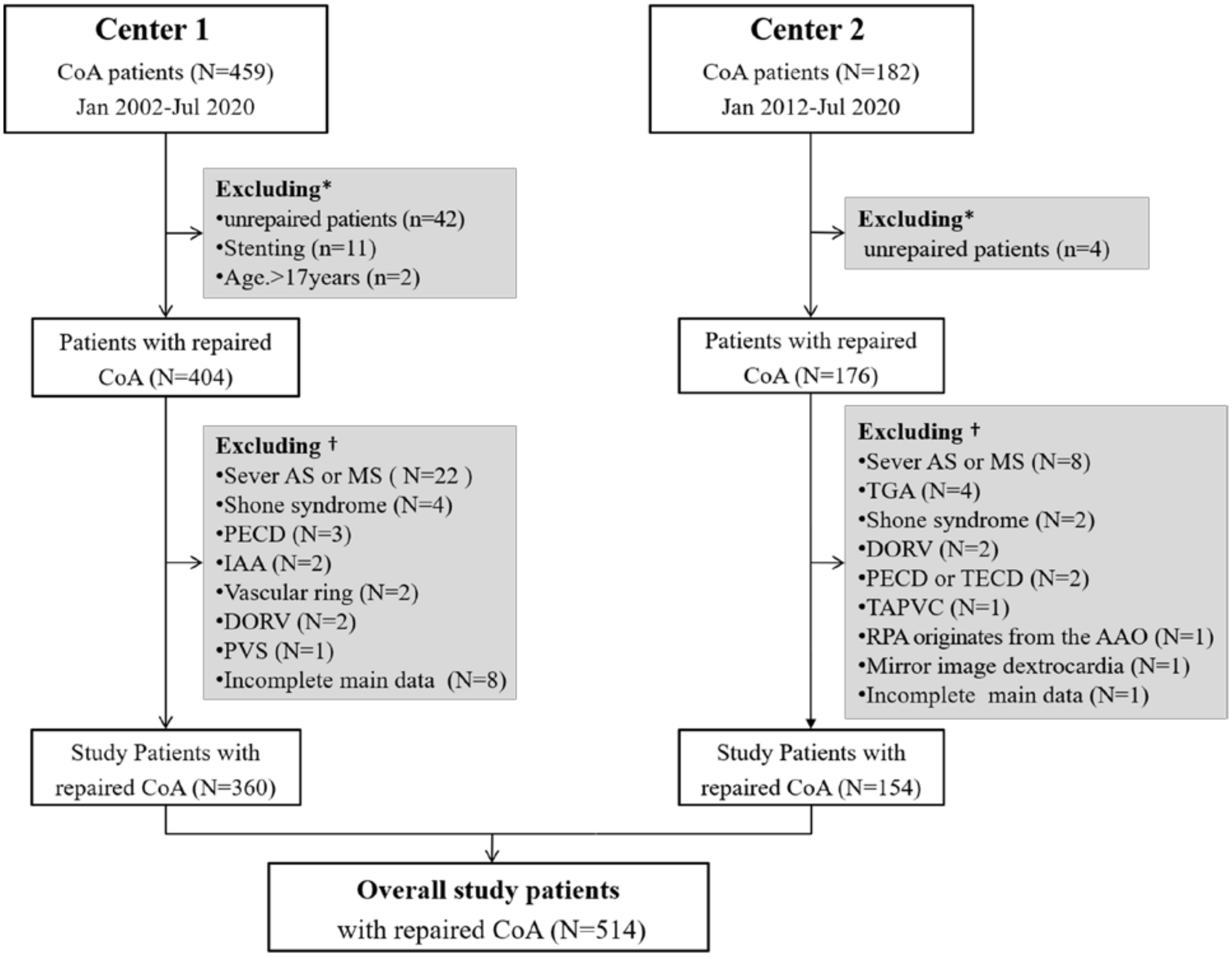

Supplementary Figure 2. Flow chart of the inclusion and exclusion processes. * Step1: to including patients with repaired CoA by surgery with an age ≤17 years; †Step2: to excluding patients with complicated co-morbidities that may independently affect cardiovascular function or incomplete main data. CoA, coarctation of aorta; AS, aortic stenosis; MS, mitral stenosis; PECD, partial endocardial cushion defect; IAA, interrupted aortic arch; DORV, double outlet of right ventricle; PVS, pulmonary vein stenosis; TGA, transposition of great arteries; TECD, total endocardial cushion defect; TAPVC, total anomalous pulmonary venous drainage; RPA, right pulmonary artery; AAO, ascending aorta.

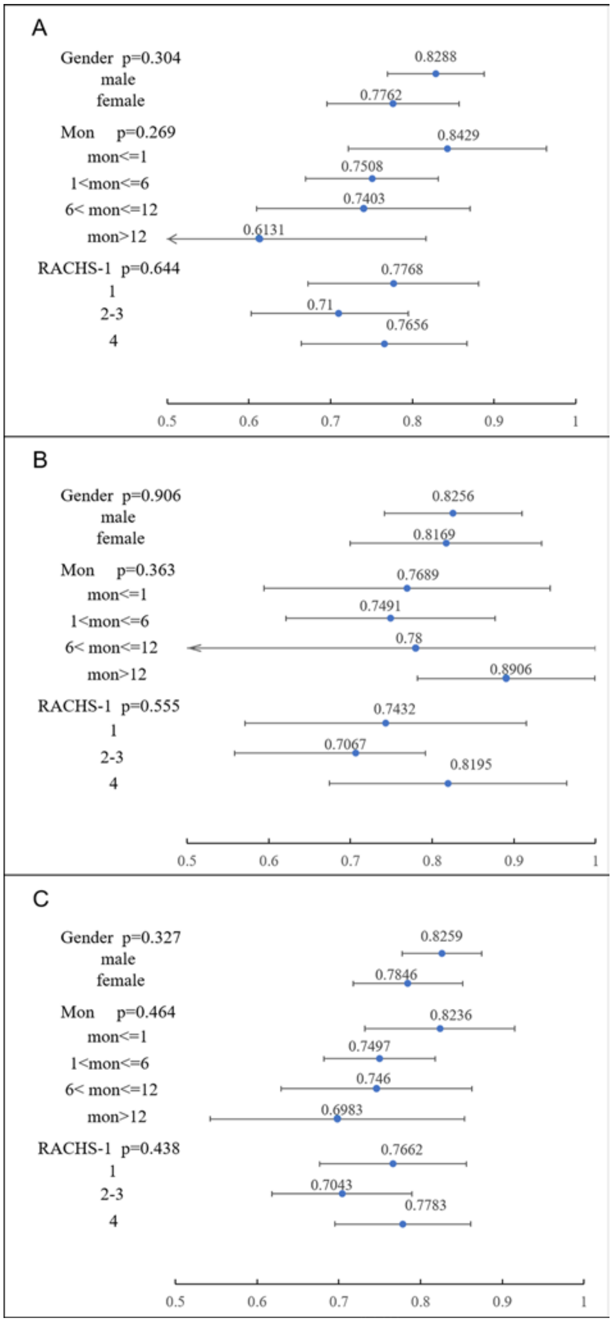

Supplementary Figure 3. Forest plot of the subgroup analysis by gender, age (months), and RACHS-1 method in the discovery set **(A)**, validation set **(B)** and overall population **(C)** respectively. Comparisons of the AUCs among the subgroups are consistent with each other respectively (p > 0.05). 95% confidence interval (CI) is indicated by line length. RACHS-1, Risk Adjustment for Congenital Heart Surgery.

References

1. Part IV – the dictionary of definitions of complications associated with the treatment of patients with congenital cardiac disease. Cardiol Young. (2008) 18 (Suppl 2): 282-530.doi: 10.1017/S1047951108003351

2. Pellicori P, Cleland JGF, Zhang J, Kallvikbacka-Bennett A, Urbinati A, Shah P, et al. Cardiac dysfunction,congestion and loop diuretics: their relationship to prognosis in Heart Failure. Cardiovasc Drugs Ther. (2016) 30(6):599-609. doi: 10.1007/s10557-016-6697-7.

3.Dickstein K, Cohen-Solal A, Filippatos G, McMurray JJ, Ponikowski P, Poole-Wilson PA, et al. ESC Guidelines for the diagnosis and treatment of acute and chronic heart failure 2008. The Task Force for the Diagnosis and Treatment of Acute and Chronic Heart Failure 2008 of the European Society of Cardiology. Developed in collaboration with the Heart. Eur J Heart Fail. (2008)10(10):933-89. doi: 10.1016/j.ejheart.2008.08.005

4. Oliver JM, Gallego P, Gonzalez AE, Sanchez-recalde A, Bret M, Aroca A. Pulmonary hypertension in young adults with repaired coarctation of the aorta: An unrecognised factor associated with premature mortality and heart failure. Int J Cardiol. (2014) 174(2):324-9. doi: 10.1016/j.ijcard.2014.04.060

5. Jain A, McNamara PJ. Persistent pulmonary hypertension of the newborn: Advances in diagnosis and treatment. Semin Fetal Neonatal Med. (2015) 20(4):262-71. doi: 10.1016/j.siny.2015.03.001

6. Lee MG, Kowalski R, Galati JC, Cheung MM, Jones B, Koleff J, et al. Twenty-four-hour ambulatory blood pressure monitoring detects a high prevalence of hypertension late after coarctation repair in patients with hypoplastic arches. J Thorac Cardiovasc Surg. (2012) 144(5):1110-6. doi: 10.1016/j.jtcvs.2012.08.013

7. Devereux RB, Alonso DR, Lutas EM, Gottlieb GJ, Campo E, Sachs I, et al. Echocardiographic assessment of left ventricular hypertrophy: Comparison to necropsy findings. Am J Cardiol. (1986) 57(6): 450-8.doi: 10.1016/0002-9149(86)90771-x

8. Khoury PR, Mitsnefes M, Daniels SR, Kimball TR. Age-specific reference intervals for indexed left ventricular mass in children. J Am Soc Echocardiogr. (2009) 22(6):709-14. doi: 10.1016/j.echo.2009.03.003

9. Flynn JT, Falkner BE. New clinical practice guideline for the management of highblood pressure in children and adolescents. Hypertension. (2017) 70(4):683-686. doi:10.1161/ HYPERTENSIONAHA.117.10050

10. Falkner B, Daniels SR. Summary of the fourth report on the diagnosis, evaluation, and treatment of high blood pressure in children and adolescents. Hypertension. (2004) 44(4):387-8. doi: 10.1161/01.HYP.0000143545.54637.af

11.Hanevold C, Waller J, Daniels S, Portman R, Sorof J; International Pediatric Hypertension Association. The effects of obesity, gender, and ethnic group on left ventricular hypertrophy and geometry in hypertensive children: a collaborative study of the international pediatric hypertension association. Pediatrics. (2004) 113(2):328-33. doi: 10.1542/peds.113.2.328.

12.Daniels SR, Meyer RA, Liang YC, Bove KE. Echocardiographically determined left ventricular mass index in normal children, adolescents and young adults. J Am Coll Cardiol. (1988) 12(3):703-8. doi: 10.1016/s0735-1097(88)80060-3

13. Lurie PR. Changing concepts of endocardial fibroelastosis. Cardiol Young. (2010) 20(2):115-23. doi: 10.1017/S1047951110000181

14. Karatza A, Mylonas KS, Tzifa A. Left ventricular non-compaction in a child with bicuspid aortic valve and aortic coarctation. Cardiology in the Young. (2019) 29(9):1208-1210. doi: 10.1017/S1047951119001707

15. Jenkins KJ, Gauvreau K, Newburger JW, Spray TL, Moller JH, Iezzoni LI. Consensus-based method for risk adjustment for surgery for congenital heart disease. Thorac Cardiovasc Surg. (2002) 123(1):110-8. doi: 10.1067/mtc.2002.119064

16. Clarke D, Jacobs J, Comas J, et al. The Aristotle score: a complexity-adjusted method to evaluate surgical results. Eur J Cardiothorac Surg. (2004) 25(6):911-24. doi: 10.1016/j.ejcts.2004.03.027

17. Brien SMO, Clarke DR, Jacobs JP, Jacobs ML, Lacour-Gayet FG, Pizarro C, et al. An empirically based tool for analyzing mortality associated with congenital heart surgery. J Thorac Cardiovasc Surg. (2009) 138(5): 1139-53.doi: 10.1016/j.jtcvs.2009.03.071

18. Jacobs JP, Brien SMO, PasqualiSK, Jacobs ML, Lacour-Gayet FG, Tchervenkov CI, et al. Variation in outcomes for risk-stratified pediatric cardiac surgical operations: an analysis of the STS Congenital Heart Surgery Database. Ann Thorac Surg. (2012) 94(2):564-71; discussion 571-2. doi: 10.1016/j.athoracsur.2012.01.105.

19. Jacobs ML, Brien SMO, Jacobs JP, Mavroudis C, Lacour-Gayet F, Pasquali SK, et al. An empirically based tool for analyzing morbidity associated with operations for congenital heart disease. J Thorac Cardiovasc Surg. (2013) 145(4):1046-1057.e1. doi: 10.1016/j.jtcvs.2012.06.029
